# Visceral adiposity index is associated with arterial stiffness in hypertensive adults with normal-weight: the china H-type hypertension registry study

**DOI:** 10.1186/s12986-021-00617-5

**Published:** 2021-10-09

**Authors:** Junpei Li, Jian Zhu, Ziheng Tan, Yun Yu, Linfei Luo, Wei Zhou, Linjuan Zhu, Tao Wang, Tianyu Cao, Lishun Liu, Huihui Bao, Xiao Huang, Xiaoshu Cheng

**Affiliations:** 1 Department of Cardiology, Nanchang University Second Affiliated Hospital, No. 1 Minde Road, Nanchang, 330006 Jiangxi China; 2 Qiukou Health Center, Wuyuan, China; 3 Center for Prevention and Treatment of Cardiovascular Diseases, Nanchang University Second Affiliated Hospital, Nanchang, Jiangxi China; 4 Biological Anthropology, University of California, Santa Barbara, California USA; 5 Beijing Advanced Innovation Center for Food Nutrition and Human Health, College of Food Science and Nutritional Engineering, China Agricultural University, Beijing, China

**Keywords:** Visceral adiposity index, Brachial-ankle pulse wave velocity, Body mass index, Normal weight, Hypertension

## Abstract

**Background:**

Limited information is available on arterial stiffness risk among hypertensive patients with metabolically abnormal but normal weight. Visceral adiposity index (VAI) is a novel indicator for visceral fat mass and metabolism, however, whether can be used to assessed arterial stiffness in a normal-weight population remains unclear. The goal of this study was to examine the independent association of VAI with arterial stiffness in normal-weight hypertensive patients.

**Methods:**

3258 participants recruited from the China H-type Hypertension Registry Study. VAI value was calculated using sex-specific equations. High arterial stiffness was defined as baPWV ≥ 18 m/s. Multivariable regression analysis was used to identify the association of VAI with baPWV and high arterial stiffness.

**Results:**

Of participants, 50.5% (1644) were males, the mean age was 65.5 (SD, 9.1) years. Mean VAI and baPWV were 2.0 (SD, 2.3) and 18.2 (SD, 3.9) m/s, respectively. For each unit increase of lg VAI in multivariable regression analysis, there was a 1.05 m/s increase in baPWV (95% CI 0.67, 1.43) and a 2.13-fold increase in the risk of high arterial stiffness (95% CI 1.59, 2.86). In all models, the VAI was consistently and significantly associated with baPWV after adjustment for different confounders. High VAI levels were stably associated with baPWV in all subgroups.

**Conclusions:**

We found positive association of VAI with baPWV and high arterial stiffness in normal-weight adults with hypertension. The establishment of this association could help the arterial stiffness risk stratification in normal-weight hypertensive populations, who are frequently overlooked in preventing cardiovascular disease.

**Supplementary Information:**

The online version contains supplementary material available at 10.1186/s12986-021-00617-5.

## Introduction

Appropriate large arterial elasticity exerts a buffering effect to protect the microcirculation and main organ from harmful fluctuations in pressure and pulsatile flow by limiting arterial pulsatility. Nevertheless, reduced arterial elasticity and elevated arterial stiffness is the most common cause of chronic common cardiovascular diseases and is frequently associated with morbidity and mortality globally [1–3]. A meta-analysis of 17 longitudinal studies with 15,877 subjects for a mean of 7.7 years followed-up concluded that per 1 m/s increase in aortic pulse wave velocity (PWV), a quantitative index of arterial stiffness, corresponded to adjusted risk in total cardiovascular events, cardiovascular mortality, and all-cause mortality with respective increases of 14%, 15%, and 15% [4]. Arterial stiffness is the leading cause of elevated blood pressure and hypertension, which in turn predisposes individuals to elevated arterial stiffness [5]. Data from 90 countries estimated that over 1.3 billion (31.1%) adults worldwide living with hypertension in 2010 [6, 7]. According to the Report on Cardiovascular Diseases in China, approximately 270 million Chinese were living with hypertension in 2017 [8]. Framingham Offspring Study suggested that excessed weight, especially visceral adiposity deposited, is a major cause of hypertension, accounting for 65% and 78% of the risk for primary hypertension in women and men, respectively [9]. The coexistence of hypertension with increased visceral adiposity substantially contributes to the likelihood of arterial stiffness.

Visceral adiposity is the fat deposited around internal organ sites. Radiographic imaging, including computed tomography (CT) and magnetic resonance imaging (MRI), can accurately and sensitively detect the distribution and amount of visceral adiposity. However, these methods are time-consuming and costly, which limiting their use in the general population. Based on simple parameters including body mass index (BMI), waist circumference (WC), triglycerides (TG), and high-density lipoprotein cholesterol (HDL), the visceral adiposity index (VAI) is an empirical-mathematical model to indicate the visceral fat distribution and function, which shows a strong correlation with the visceral fat area (Spearman, R_s_ = 0.437, *P* = 0.025) and volume (R_s_ = 0.744, *P* < 0.001) detected by MRI [10].

Growing evidence has verified that VAI is associated with insulin resistance [11, 12], kidney dysfunction [13] and cardiovascular disease [14, 15]. Nevertheless, most of the studies mentioned above usually focused on the obese, with limited attention to normal-weight populations. An estimated 20% of the normal weight cohort was classified as metabolically obese, which involves a series of risk factors of metabolic disorders [16, 17]. Bouchi et al. investigated type 2 diabetes mellitus (T2DM) patients admitted to the Tokyo Medical and Dental University Hospital, which indicated that 7.2% of participants (N = 30) were eligible for both normal weight and visceral fat area ≥ 100 cm^2^ [18]. Therefore, it is important to know the risk of visceral adiposity in normal-weight populations. To date, though, the role of VAI as a risk evaluation tool of arterial stiffness in the hypertension population with normal weight remains poorly understood. Few investigations have further been performed to addressed potential effect modifications of the relationship between VAI and arterial stiffness. This study, as a part of the China H-type Hypertension Registry Study, was undertaken to improve the understanding of the association between VAI and arterial stiffness in the hypertensive population with normal-weight, beyond that, we sought to further explore the potential modifiers.

## Methods

### Study design and population

This is a cross-sectional baseline survey, which is part of the China H-type Hypertension Registry Study (Registration number: ChiCTR1800017274), a real-world, observational study designed to enroll and monitor cohorts of the hypertensive population with high prevalence of hyperhomocysteinemia in China, which conducted from March 2018 in Wuyuan, China. Methodological details, including inclusion and exclusion criteria of the China H-type hypertension Registery Study, have been previously published elsewhere [19]. In brief, participants ≥ 18 years of age with a diagnosis of hypertension, defined as systolic blood pressure (SBP) ≥ 140 mm Hg or diastolic blood pressure (DBP) ≥ 90 mm Hg in the sitting position after 10 min of rest or use of antihypertensive treatment, were eligible for inclusion. Participants who could not accept the long-term follow-up visit due to objective reasons or those living with severe neurological or psychiatric conditions resulting in an inability to demonstrate informed consent were excluded. The study protocol was approved by the Ethics Committee of the Institute of Biomedicine, Anhui Medical University, and performed in accordance with the Declaration of Helsinki. All participants provided written informed consent after reading a statement that explained the purpose of the study.

Analysis was restricted to the subset of participants who had baPWV data at the time of recruitment (n = 5233). Two participants were missing BMI data. Participants with underweight (BMI < 18.5 kg/m^2^, n = 409), overweight and obese (BMI 25 to < 30 kg/m^2^ and ≥ 30 kg/m^2^, n = 1564) categorized according to the World Health Organization were excluded. Ultimately, 3258 participants were suitable for the final analysis. The flow of the screening process was detailed in Additional file 1: Figure S1.

### Clinical characteristics

At recruitment, socio-demographic, medical history, and drug use data were collected at the health center during an interview by trained research staff who followed the standard operating procedure of the study. Participants were seated for blood pressure measurements under resting conditions. The blood pressure was measured by a calibrated electronic sphygmomanometer (OMRON; Dalian, China) in both arms at recruitment and thereafter repeated four times in the arm that showed the higher blood pressure, the average blood pressure value was taken. Waist circumference, height, and weight were collected without shoes and heavy clothes and measured by the medical scale. Body mass index (BMI) was calculated by the formula BMI = weight (kg)/square of height (m^2^). Cigarette smoking, including never, former, and current smoking, was defined as smoking ≥ 1 cigarette per day for 1 year or more or a cumulative smoking amount ≥ of 360 cigarettes per year. Alcohol consumption was defined as drinking an average of at least 2 or more times a week over a year.

### Laboratory assays

All blood samples were collected in the fasting state, then flash processed and frozen. Serum fasting blood glucose (FBG), total cholesterol, TG, HDL, creatinine, and homocysteine levels were assayed using automatic clinical analyzers (reagents from Guangdong Biaojia Biotechnology Co., Ltd., using Beckman Coulter AU680). The estimated glomerular filtration rate (eGFR) was calculated using the equation of Chronic Kidney Disease Epidemiology Collaboration (CKD-EPI). We defined diabetes in this study as self-reported diabetes or use of antidiabetic drugs or FBG concentration ≥ 7.0 mmol/L.

### Calculation of VAI

VAI was calculated by using the following sex-specific equations. In the equations, the unit of WC is cm; BMI is kg/m^2^; TG and HDL are mmol/L.

$${\text{VAI}}_{{{\text{males}}}} = \frac{WC}{{39.68{ } + { }\left( {1.88{ } \times {\text{BMI}}} \right)}} \times \left( {\frac{{{\text{TG}}}}{1.03}} \right) \times \left( {\frac{1.31}{{{\text{HDL}}}}} \right)$$

$${\text{VAI}}_{{{\text{females}}}} = \frac{WC}{{36.58{ } + { }\left( {1.89{ } \times {\text{ BMI}}} \right)}} \times \left( {\frac{{{\text{TG}}}}{0.813}} \right) \times \left( {\frac{1.52}{{{\text{HDL}}}}} \right)$$

### Brachial-ankle pulse wave velocity (BaPWV) measurements

BaPWV was automatically measured by Omron Colin BP-203RPE III device (Omron Health Care, Kyoto, Japan). The participants were positioned supine after at least 5 min of bed rest, with both the arms and ankles exposed and fully extended. Bilateral brachial and ankles arterial pressure waveforms were recorded by extremities cuffs connected to a plethysmographic sensor and oscillometric pressure sensor. The baPWV was automatically computed as the brachium and ankle transmission distance divided by the time interval between the wavefront of the brachial waveform and that of the ankle waveform (La − Lb)/Tba, with the highest baPWV value being used.

### Statistical analysis

Continuous data are presented as means with standard deviations (SD) and categorical variables are given as frequencies and percentages. The baseline characteristics of the study population were presented by quartile groups of VAI. Group comparison was performed with variance (ANOVA) or Chi-squared tests. High baPWV value of 18 m/s was widely used as a cut-off threshold to identify for high-risk of cardiovascular events in the Asian population [20, 21], thus, we used baPWV value greater than 18 m/s as define of high arterial stiffness. The VAI was not normally distributed; the variable was log-transformed, and the transformed version was used for analysis when VAI was treated as continuous. Independent association of VAI with baPWV and high arterial stiffness was done by multivariable linear regression models and multivariable regression logistic regression models. These covariates were included in the adjusted regression models when satisfying one of the following conditions: (1) the regression coefficient P-value for covariates on outcome variable < 0.10, or (2) the incorporation of covariates in the model causes a more than 10% change in regression coefficients, or (3) covariates as potential confounders based on prior literature. Crude model did not include any covariates; Model I adjusted for sex and age; Model II further adjusted SBP; DBP; years of hypertension; cigarette smoking; alcohol consumption; homocysteine; total cholesterol; eGFR; diabetes; self-reported stroke and coronary heart disease; antihypertensive drugs; lipid-lowering drugs. The generalized additive model with the smoothing curve (penalized spline method) was used to analyze the dose–response association of VAI with baPWV and high arterial stiffness. Finally, further stratified analyses by subgroups including sex, age, SBP, DBP, years of hypertension, cigarette smoking, alcohol consumption, homocysteine, total cholesterol, eGFR, diabetes, stroke, antihypertensive drugs, and lipid-lowering drugs were also explored by multivariable linear regression models to test for consistency of results. Tests for interaction were performed by using a likelihood ratio test to compare models with and without interaction terms.

All analyses were done using Empower (R) statistical software (www. empowerstats.com; X&Y Solutions, Inc., Boston, MA) and the statistical package R (http://www.r-project.org). A 2-tailed P < 0.05 was considered statistically significant.

## Results

### Participant characteristics

A total of 3258 hypertensive participants of the China H-type Hypertension Registry Study with normal weight were included in the final analysis. Of them, 50.5% (1644) were males, the mean age of the participants was 65.5 (SD, 9.1) years. Mean VAI and baPWV were 2.0 (SD, 2.3) and 18.2 (SD, 3.9) m/s, respectively. Characteristics of the study participants by VAI quartiles were shown in Table 1. Participants with higher VAI (≥ 2.35) seemed to be younger, females, have lower levels of homocysteine, HDL, as well as a lower proportion of current cigarette smoking and alcohol consumption than other groups, but more likely to have higher BMI, waist circumference, fasting glucose, total cholesterol, triglyceride, eGFR levels, and the prevalence of diabetes.

**Table 1:** Characteristics of the study population by quartile groups of VAI

Characteristics	Visceral adiposity index				P value
	Q1: < 0.86	Q2: ≥ 0.86, < 1.41	Q3: ≥ 1.41, < 2.35	Q4: ≥ 2.35	
N	796	832	815	815	
Age, years	66.9 ± 9.0	66.4 ± 9.2	65.3 ± 8.8	63.5 ± 9.0	< 0.001
Male, n (%)	646 (81.2)	476 (57.2)	310 (38.0)	212 (26.0)	< 0.001
BMI, kg/m^2^	21.3 ± 1.7	21.9 ± 1.7	22.3 ± 1.7	22.8 ± 1.6	< 0.001
Waist circumference, cm	76.3 ± 6.3	78.7 ± 6.5	80.5 ± 6.4	82.5 ± 5.9	< 0.001
BaPWV, m/s	17.9 ± 3.7	18.1 ± 4.0	18.5 ± 3.9	18.5 ± 4.0	0.002
Visceral adiposity index	0.6 ± 0.2	1.1 ± 0.2	1.8 ± 0.3	4.4 ± 3.5	< 0.001
High arterial stiffness, n (%)	329 (41.3)	360 (43.3)	400 (49.1)	404 (49.6)	< 0.001
SBP, mm Hg	147.4 ± 18.6	147.7 ± 17.6	147.1 ± 17.8	148.2 ± 17.1	0.680
DBP, mm Hg	88.0 ± 11.0	88.4 ± 10.8	87.6 ± 10.4	88.5 ± 10.9	0.308
Years of hypertension, years	6.6 ± 7.1	7.2 ± 7.8	7.2 ± 7.4	6.9 ± 7.0	0.380
*Laboratory tests*					
Homocysteine, μmol/L	19.9 ± 12.8	20.0 ± 13.5	18.4 ± 11.4	16.7 ± 7.9	< 0.001
Fasting glucose, mmol/L	5.8 ± 1.2	5.9 ± 1.3	6.1 ± 1.4	6.5 ± 1.9	< 0.001
Total cholesterol, mmol/L	4.9 ± 1.0	5.0 ± 1.1	5.2 ± 1.1	5.2 ± 1.2	< 0.001
Triglyceride, mmol/L	0.8 ± 0.2	1.2 ± 0.3	1.6 ± 0.4	2.9 ± 1.5	< 0.001
HDL, mmol/L	1.8 ± 0.4	1.6 ± 0.3	1.4 ± 0.3	1.2 ± 0.3	< 0.001
eGFR, ml/min/1.73m^2^	84.6 ± 19.4	84.8 ± 19.0	85.6 ± 19.1	87.2 ± 19.6	0.032
*Cigarette smoking, n (%)*					< 0.001
Never	245 (30.8)	390 (46.9)	510 (62.6)	544 (66.7)	
Former	200 (25.1)	171 (20.6)	121 (14.8)	106 (13.0)	
Current	351 (44.1)	271 (32.6)	184 (22.6)	165 (20.2)	
Alcohol consumption, n (%)					< 0.001
Never	336 (42.2)	508 (61.1)	573 (70.3)	618 (75.8)	
Former	134 (16.8)	126 (15.1)	79 (9.7)	75 (9.2)	
Current	326 (41.0)	198 (23.8)	163 (20.0)	122 (15.0)	
*Medical history, n (%)*					
Diabetes	85 (10.7)	107 (12.9)	140 (17.2)	216 (26.5)	< 0.001
Stroke	57 (7.2)	67 (8.1)	75 (9.2)	63 (7.7)	0.489
CHD	47 (5.9)	59 (7.1)	66 (8.1)	52 (6.4)	0.328
Antihypertensive drugs, n (%)	440 (55.3)	502 (60.3)	521 (63.9)	506 (62.1)	0.003
Lipid-lowering drugs, n (%)	21 (2.6)	26 (3.1)	26 (3.2)	33 (4.0)	0.449
Antidiabetic drugs, n (%)	18 (2.3)	26 (3.1)	31 (3.8)	57 (7.0)	< 0.001

Continuous data are presented as means with standard deviations (SD) and categorical variables are given as frequencies and percentages

VAI, visceral adiposity index; baPWV, brachial-ankle pulse wave velocity; BMI, body mass index; SBP, systolic blood pressure; DBP, diastolic blood pressure; HDL, high density lipoprotein eGFR, estimate the glomerular filtration rate; CHD, coronary heart disease

### Associations of VAI with baPWV and high arterial stiffness

**Fig. 1:**
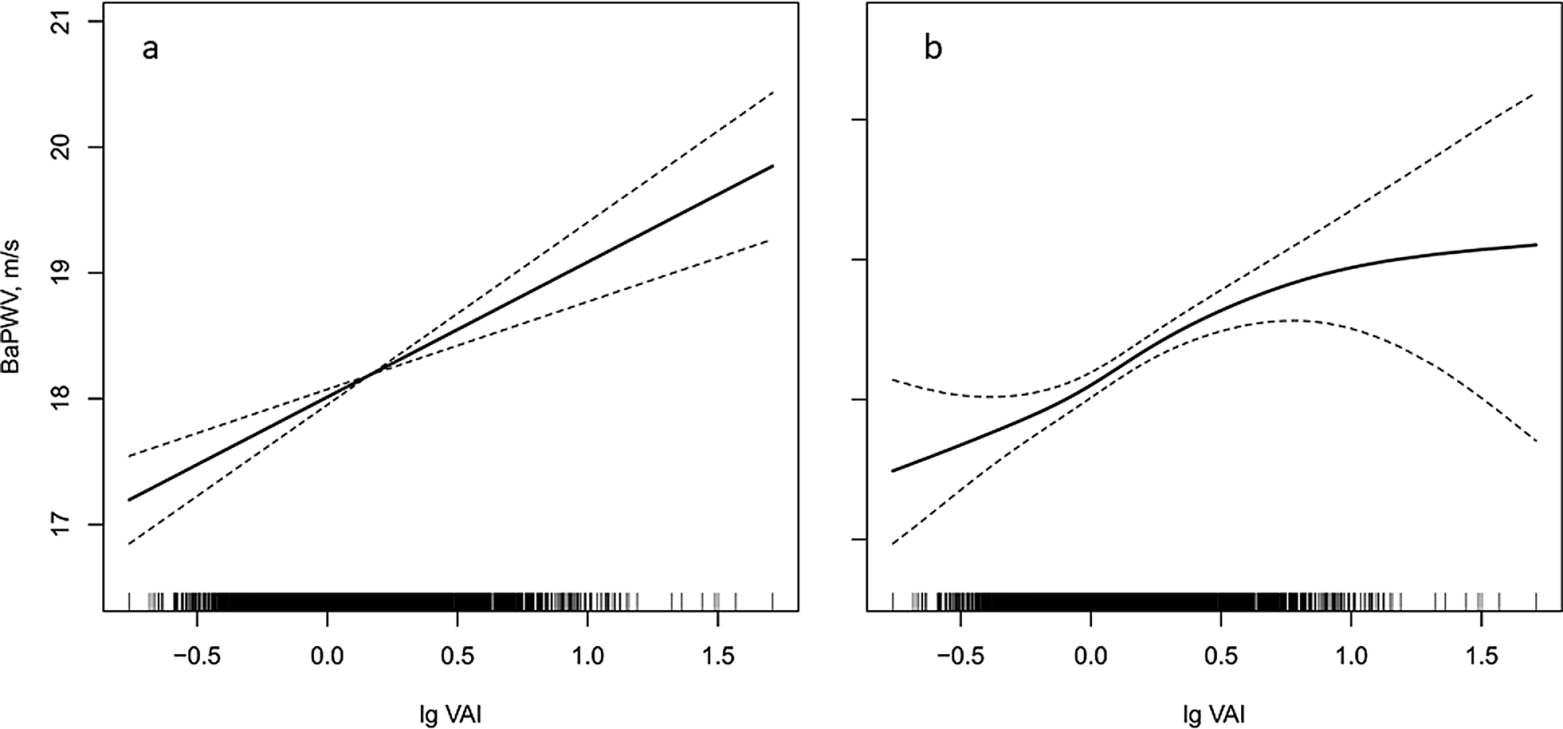
The associations of lg VAI with baPWV and high arterial stiffness in normal-weight adults with hypertension. **a** lg VAI and baPWV; **b** lg VAI and high arterial stiffness. Adjusted for sex, age, systolic blood pressure; diastolic blood pressure; years of hypertension; cigarette smoking; alcohol consumption; homocysteine; total cholesterol; eGFR; diabetes; self-reported stroke and coronary heart disease; antihypertensive drugs; lipid-lowering drugs. VAI, visceral adiposity index; baPWV, brachial-ankle pulse wave velocity; eGFR, estimate the glomerular filtration rate

Smoothing curve fitted the significant positive association of lg VAI with baPWV and high arterial stiffness (Fig. 1a and b). For each unit increase of lg VAI, there was 1.05 m/s increase in baPWV (95% CI 0.67, 1.43) and 2.13-fold increase in risk of high arterial stiffness (95% CI 1.59, 2.86). In all models, the VAI was consistently and significantly associated with baPWV after adjustment for different confounders. When VAI was treated as quartiles, the adjusted β for participants in the third and fourth quartiles were 0.61 (95% CI 0.28, 0.93) and 0.74 (95% CI 0.40, 1.08), respectively, compared with those in first quartile (P for trend < 0.001) (Table 2). Additionally, the risk of high arterial stiffness was generally greater in patients with VAI levels in quartile 3 (OR, 1.59; 95% CI 1.23, 2.04) and 4 (OR, 1.76; 95% CI 1.35, 2.29) than those with patients in quartile 1 (P for trend < 0.001) among participants with normal-weight (Table 3). Compared with participants in lower two quartiles, a greater risk of high arterial stiffness was presented among those in upper two quartiles (OR, 1.59; 95% CI 1.32, 1.91).

**Table 2:** The association between lg VAI and baPWV in normal-weight adults with hypertension

Visceral adiposity index	N	Mean ± SD	Crude model		Model I		Model II	
			β (95%CI)	P-value	β (95%CI)	P-value	β (95%CI)	P-value
Per unit increase in lgVAI	3258	18.2 ± 3.9	0.91 (0.49, 1.32)	< 0.001	1.32 (0.90, 1.74)	< 0.001	1.05 (0.67, 1.43)	< 0.001
*Quartile*								
Q1: < 0.86	796	17.9 ± 3.7	Ref.(0)		Ref.(0)		Ref.(0)	
Q2: 0.86– < 1.41	832	18.1 ± 4.0	0.18 (-0.20, 0.56)	0.345	0.15 (-0.20, 0.50)	0.415	0.09 (-0.22, 0.40)	0.562
Q3: 1.41– < 2.35	815	18.5 ± 3.9	0.61 (0.23, 0.99)	0.002	0.64 (0.28, 1.01)	0.001	0.61 (0.28, 0.93)	< 0.001
Q4: ≥ 2.35	815	18.5 ± 4.0	0.60 (0.22, 0.98)	0.002	0.89 (0.51, 1.27)	< 0.001	0.74 (0.40, 1.08)	< 0.001
P for trend				< 0.001		< 0.001		< 0.001
*Categorical*								
Q1,2: < 1.41	1628	18.0 ± 3.9	Ref.(0)		Ref.(0)		Ref.(0)	
Q3,4: ≥ 1.41	1630	18.5 ± 3.9	0.51 (0.24, 0.78)	< 0.001	0.68 (0.41, 0.94)	< 0.0001	0.62 (0.38, 0.85)	< 0.0001

Crude model adjusted for: none; Model I adjusted for: sex; age; Model II adjusted for: sex; age; systolic blood pressure; diastolic blood pressure; years of hypertension; cigarette smoking; alcohol consumption; homocysteine; total cholesterol; estimated glomerular filtration rate; diabetes; relf-reported stroke and coronary heart disease; antihypertensive drugs; lipid-lowering drugs

VAI, visceral adiposity index; baPWV, brachial-ankle pulse wave velocity

**Table 3:** The association between lg VAI and high arterial stiffness in normal-weight adults with hypertension

Visceral adiposity index	Events (%)	Crude model		Model I		Model II	
		OR (95% CI)	P-value	OR (95% CI)	P-value	OR (95% CI)	P-value
Per unit increase in lgVAI	1493 (45.8)	1.60 (1.29, 1.98)	< 0.001	2.23 (1.72, 2.89)	< 0.001	2.13 (1.59, 2.86)	< 0.001
*Quartile*							
Q1: < 0.86	329 (41.3)	1		1		1	
Q2: 0.86– < 1.41	360 (43.3)	1.08 (0.89, 1.32)	0.429	1.09 (0.88, 1.35)	0.450	1.08 (0.85, 1.38)	0.519
Q3: 1.41- < 2.35	400 (49.1)	1.37 (1.12, 1.67)	0.002	1.51 (1.20, 1.89)	< 0.001	1.59 (1.23, 2.04)	< 0.001
Q4: ≥ 2.35	404 (49.6)	1.40 (1.15, 1.70)	0.001	1.79 (1.42, 2.27)	< 0.001	1.76 (1.35, 2.29)	< 0.001
P for trend			< 0.001		< 0.001		< 0.001
*Categorical*							
Q1,2: < 1.41	689 (42.3)	1		1		1	
Q3,4: ≥ 1.41	804 (49.3)	1.33 (1.16, 1.52)	< 0.001	1.56 (1.32, 1.83)	< 0.001	1.59 (1.32, 1.91)	< 0.001

Crude model adjusted for: none; Model I adjusted for: sex; age; Model II adjusted for: sex; age; systolic blood pressure; diastolic blood pressure; years of hypertension; cigarette smoking; alcohol consumption; homocysteine; total cholesterol; estimated glomerular filtration rate; diabetes; relf-reported stroke and coronary heart disease; antihypertensive drugs; lipid-lowering drugs

VAI, visceral adiposity index; baPWV, brachial-ankle pulse wave velocity

### Subgroup analyses by potential effect modifiers

**Fig. 2:**
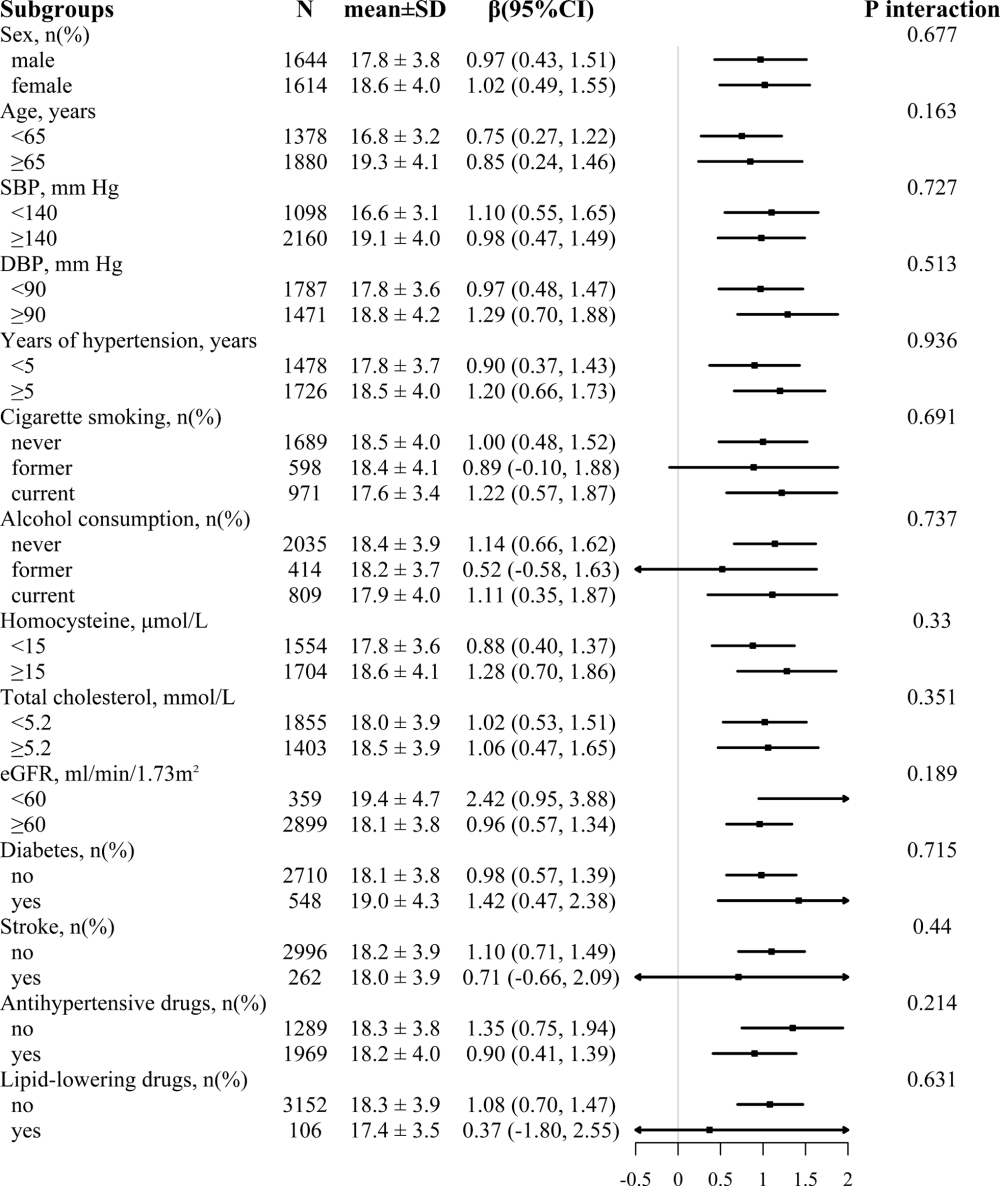
Stratified analyses of VAI for association with baPWV among normal-weight adults with hypertension. Boxes denote β, lines represent 95% CI. Adjusted, if not stratified, for sex, age, systolic blood pressure; diastolic blood pressure; years of hypertension; cigarette smoking; alcohol consumption; homocysteine; total cholesterol; eGFR; diabetes; self-reported stroke and coronary heart disease; antihypertensive drugs; lipid-lowering drugs. VAI, visceral adiposity index; baPWV, brachial-ankle pulse wave velocity; eGFR, estimate the glomerular filtration rate

Stratified analysis was carried out to assess the stability in various subgroups (Fig. 2). Subsequent results showed no significant interaction in the association of lg VAI with baPWV among the following subgroups, including sex (male vs. female, *P* for interaction = 0.677), age (< 65 vs. ≥ 65 years, *P* for interaction = 0.163), SBP (< 140 vs. ≥ 140 mm Hg, *P* for interaction = 0.727), DBP (< 90 vs. ≥ 90 mm Hg, *P* for interaction = 0.513), years of hypertension (< 5 vs. ≥ 5 years, *P* for interaction = 0.936), alcohol consumption (never vs. former vs. current, *P* for interaction = 0.691), alcohol consumption (never vs. former vs. current, *P* for interaction = 0.737), homocysteine levels (< 15 vs. ≥ 15 μmol/L, *P* for interaction = 0.330), total cholesterol (< 5.2 vs. ≥ 5.2 mmol/L, *P* for interaction = 0.351), eGFR (< 60 vs. ≥ 60 ml/min/1.73m^2^, *P* for interaction = 0.189), diabetes (no vs. yes, *P* for interaction = 0.715), stroke (no vs. yes, *P* for interaction = 0.440), use of antihypertensive drugs (no vs. yes, *P* for interaction = 0.214) and lipid-lowering drugs (no vs. yes, *P* for interaction = 0.631), tested by linear regression analysis.

## Discussion

The findings presented in this study were the first to examine the independently and stably association of VAI with baPWV and the risk of high arterial stiffness in hypertensive adults with normal-weight.

Some researchers had reported relevant studies, but multiple studies had focused on general or diabetes-related populations. Choi et al. reported that VAI was positively associated with arterial stiffness, represented by the mean baPWV, in both Korean males and females who underwent a routine health checkup [22]. This result was supported by study performed in community-dwelling individuals over 40 years of age in China [23]. A cross-sectional study recruited 97 postmenopausal females with T2DM examined that increased visceral fat were independently associated with increased arterial stiffness in postmenopausal females [24]. Among non-industrial workers aged 25 to 55, Nakagomi and his groups found a stronger association between VAI and baPWV in females than males [25]. But Morigami et al. demonstrated visceral fat area and subcutaneous fat area were associated with arterial wall stiffness only in males with T2DM but not in females [26]. The differences in sample size, inconsistent definitions, age range of study population and participants screening methods may partially explain why these studies' conclusions were inconsistencies. Additionally, the presence of the general obesity population may still confound the impact of VAI on arterial stiffness, although most of these studies had adjusted for BMI to rule out or minimize the influence of obesity. However, the evidence that follows with normal-weight population was still limited. The study by Bouchi et al. showed compared to patients with normal-weight and visceral fat area < 100 cm^2^, those with normal weight and visceral fat area ≥ 100 cm^2^ were significantly associated with increased baPWV even after adjustment for covariates which could affect arterial stiffness [18]. Our study was possibly the first to identified and validated the dose-dependent association of VAI with baPWV and the risk of high arterial stiffness in normal-weight individuals with hypertension. Consistent results were observed in stratified analysis, there was no significant interaction between males and females. Thus, our findings indicated that VAI could be used as a simple and reliable alternative marker for identifying the risk of high arterial stiffness in normal-weight adults with hypertension.

Chronic inflammation and insulin resistance were possible mechanisms linking VAI and arterial stiffness. Visceral adipocyte was the primary source of inflammatory cytokines and adipokines [27, 28], which may elicit reactive oxygen species (ROS) production in arterioles. The inflammatory cytokines and ROS work together, leading to reduced production and increased consumption of nitric oxide (NO) [29]. Various lines of research had demonstrated that reduced NO concentrations were significantly associated with increased risk of arterial stiffness [30, 31]. Further, the "portal theory" showed that the accumulation of visceral adiposity and linked chronic inflammation also promoted insulin resistance by producing free fatty acids and accumulating in the liver [32, 33]. In this study, higher FBG levels were observed in participants with fourth quartile VAI. Through the up-regulate activity of the renin–angiotensin–aldosterone system, insulin resistance and chronic hyperglycemia promoted hypertrophy and fibrosis of the vascular wall, consequently leading to stiffening of the artery [34].

There were several limitations of our study that deserve further mention. First, the cross-sectional study design, in which the variables were measured at the same time point, makes it difficult to infer temporal relationships or causality and assess the predictive ability of VAI for arterial stiffness. Second, the baPWV test at recruitment was voluntary and it might, therefore, restrict the representativeness of the sample included. The mean age of the participants in our study was 65.5 (SD, 9.1) years and 45.8% of participants were high arterial stiffness with baPWV ≥ 18 m/s, thus, the study conclusions may not be generalizable to the normal-weight younger populations, who had a better vascular function. Moreover, we failed to determine changes in the recent weight of participants, which means overweight or underweight participants might be misclassified to normal-weight due to the recent weight changes. Finally, although an extensive list of possible confounding factors was adjusted in the multivariable regression analysis, unrecognized residual confounding variables may still remain. Absence of imaging data in the present study, we were unable to adjust for subcutaneous adipose tissue or total adipose tissue in the model due to limited budget. Indeed, Arner et al. reported a low correlation between PWV and subcutaneous adipocyte volume (r = 0.20, *P* = 0.031), assessed by dual-X-ray absorptiometry in obese subjects. Still, the relationship became non-significant after controlling for visceral fat cell volume [35]. However, visceral adipocyte volume was strongly (*P* < 0.0001) and positively correlated with PWV. Similar results have also been reported by Han et al. [36]. Based on the above study thus, we think that non-adjustment for subcutaneous adipose tissue or total adipose tissue in the regression model might have little effect on our results.

## Conclusions

In the current study, we observed the stable and significant association of VAI with baPWV and high arterial stiffness in a dose-dependent manner among normal-weight adults with hypertension. The establishment of this association could help the arterial stiffness risk stratification in normal-weight hypertensive populations, who are frequently overlooked in preventing cardiovascular disease.

## Supplementary Information

**Additional file 1. Figure S1**. Flow chart of the study participants.

## Data Availability

The dataset used and analyzed during the current study is available from the corresponding author on reasonable request.

## References

[1] Ohkuma T, Ninomiya T, Tomiyama H, Kario K, Hoshide S, Kita Y, Inoguchi T, Maeda Y, Kohara K, Tabara Y (2017). Brachial-ankle pulse wave velocity and the risk prediction of cardiovascular disease: an individual participant data meta-analysis. Hypertension.

[2] Tomiyama H, Koji Y, Yambe M, Shiina K, Motobe K, Yamada J, Shido N, Tanaka N, Chikamori T, Yamashina A (2005). Brachial – ankle pulse wave velocity is a simple and independent predictor of prognosis in patients with acute coronary syndrome. Circ J.

[3] Sheng CS, Li Y, Li LH, Huang QF, Zeng WF, Kang YY, Zhang L, Liu M, Wei FF, Li GL (2014). Brachial-ankle pulse wave velocity as a predictor of mortality in elderly Chinese. Hypertension.

[4] Vlachopoulos C, Aznaouridis K, Stefanadis C (2010). Prediction of cardiovascular events and all-cause mortality with arterial stiffness: a systematic review and meta-analysis. J Am Coll Cardiol.

[5] Safar ME (2018). Arterial stiffness as a risk factor for clinical hypertension. Nat Rev Cardiol.

[6] Mills KT, Bundy JD, Kelly TN, Reed JE, Kearney PM, Reynolds K, Chen J, He J (2016). Global disparities of hypertension prevalence and control: a systematic analysis of population-based studies from 90 countries. Circulation.

[7] Mills KT, Stefanescu A, He J (2020). The global epidemiology of hypertension. Nat Rev Nephrol.

[8] Ma L, Wu Y, Wang W, Chen W (2018). Interpretation of the report on cardiovascular diseases in China (2017). Chin J Cardiovasc Med.

[9] Garrison RJ, Kannel WB, Stokes J, Castelli WP (1987). Incidence and precursors of hypertension in young adults: the Framingham Offspring Study. Prev Med.

[10] Amato MC, Giordano C, Galia M, Criscimanna A, Vitabile S, Midiri M, Galluzzo A, AlkaMeSy Study G. Visceral Adiposity Index: a reliable indicator of visceral fat function associated with cardiometabolic risk. Diabetes Care. 2010;33:920–2. 10.2337/dc09-1825 PMC2845052 20067971

[11] Oh JY, Sung YA, Lee HJ (2013). The visceral adiposity index as a predictor of insulin resistance in young women with polycystic ovary syndrome. Obesity (Silver Spring).

[12] Yang Y, Feng Y, Ma X, Chen K, Wu N, Wang D, Li P, Wang M, Li Q, Zhang J (2015). Visceral adiposity index and insulin secretion and action in first-degree relatives of subjects with type 2 diabetes. Diabetes Metab Res Rev.

[13] Liu M, Zhou C, Zhang Z, He P, Zhang Y, Xie D, Nie J, Liang M, Song Y, Liu C (2021). Relationship of visceral adiposity index with new-onset proteinuria in hypertensive patients. Clin Nutr.

[14] Kouli GM, Panagiotakos DB, Kyrou I, Georgousopoulou EN, Chrysohoou C, Tsigos C, Tousoulis D, Pitsavos C (2017). Visceral adiposity index and 10-year cardiovascular disease incidence: The ATTICA study. Nutr Metab Cardiovasc Dis.

[15] Yu Y, Zhang FL, Yan XL, Zhang P, Guo ZN, Yang Y (2021). Visceral adiposity index and cervical arterial atherosclerosis in northeast China: a population based cross-sectional survey. Eur J Neurol.

[16] Zheng Q, Lin W, Liu C, Zhou Y, Chen T, Zhang L, Zhang X, Yu S, Wu Q, Jin Z, Zhu Y (2020). Prevalence and epidemiological determinants of metabolically obese but normal-weight in Chinese population. BMC Public Health.

[17] Wang B, Zhuang R, Luo X, Yin L, Pang C, Feng T, You H, Zhai Y, Ren Y, Zhang L (2015). Prevalence of metabolically healthy obese and metabolically obese but normal weight in adults worldwide: a meta-analysis. Horm Metab Res.

[18] Bouchi R, Minami I, Ohara N, Nakano Y, Nishitani R, Murakami M, Takeuchi T, Akihisa M, Fukuda T, Fujita M, et al. Impact of increased visceral adiposity with normal weight on the progression of arterial stiffness in Japanese patients with type 2 diabetes. BMJ Open Diabetes Res Care. 2015;3:e000081. 10.1136/bmjdrc-2015-000081 PMC4360821 25806115

[19] Li M, Zhan A, Huang X, Hu L, Zhou W, Wang T, Zhu L, Bao H, Cheng X (2020). Positive association between triglyceride glucose index and arterial stiffness in hypertensive patients: the China H-type Hypertension Registry Study. Cardiovasc Diabetol.

[20] Munakata M (2014). Brachial-ankle pulse wave velocity in the measurement of arterial stiffness: recent evidence and clinical applications. Curr Hypertens Rev.

[21] Wakasugi S, Mita T, Katakami N, Okada Y, Yoshii H, Osonoi T, Kuribayashi N, Taneda Y, Kojima Y, Gosho M (2021). Associations between continuous glucose monitoring-derived metrics and arterial stiffness in Japanese patients with type 2 diabetes. Cardiovasc Diabetol.

[22] Choi HS, Cho YH, Lee SY, Park EJ, Kim YJ, Lee JG, Yi YH, Tak YJ, Hwang HR, Lee SH (2019). Association between new anthropometric parameters and arterial stiffness based on brachial-ankle pulse wave velocity. Diabetes Metab Syndr Obes.

[23] Yang F, Wang G, Wang Z, Sun M, Cao M, Zhu Z, Fu Q, Mao J, Shi Y, Yang T. Visceral adiposity index may be a surrogate marker for the assessment of the effects of obesity on arterial stiffness. PLoS One. 2014;9:e104365. 10.1371/journal.pone.0104365 PMC4126713 25105797

[24] Tanaka KI, Kanazawa I, Sugimoto T (2016). Reduced muscle mass and accumulation of visceral fat are independently associated with increased arterial stiffness in postmenopausal women with type 2 diabetes mellitus. Diabetes Res Clin Pract.

[25] Nakagomi A, Sunami Y, Kawasaki Y, Fujisawa T, Kobayashi Y. Sex difference in the association between surrogate markers of insulin resistance and arterial stiffness. J Diabetes Complic. 2020;34:107442. 10.1016/j.jdiacomp.2019.107442 31668590

[26] Morigami H, Morioka T, Yamazaki Y, Imamura S, Numaguchi R, Asada M, Motoyama K, Mori K, Fukumoto S, Shoji T (2016). Visceral adiposity is preferentially associated with vascular stiffness rather than thickness in men with type 2 diabetes. J Atheroscler Thromb.

[27] Nagajyothi F, Desruisseaux MS, Thiruvur N, Weiss LM, Braunstein VL, Albanese C, Teixeira MM, de Almeida CJ, Lisanti MP, Scherer PE, Tanowitz HB (2008). Trypanosoma cruzi infection of cultured adipocytes results in an inflammatory phenotype. Obesity (Silver Spring).

[28] Bastard JP, Maachi M, Lagathu C, Kim MJ, Caron M, Vidal H, Capeau J, Feve B (2006). Recent advances in the relationship between obesity, inflammation, and insulin resistance. Eur Cytokine Netw.

[29] Gunnett CA, Lund DD, McDowell AK, Faraci FM, Heistad DD (2005). Mechanisms of inducible nitric oxide synthase-mediated vascular dysfunction. Arterioscler Thromb Vasc Biol.

[30] Stewart AD, Millasseau SC, Kearney MT, Ritter JM, Chowienczyk PJ (2003). Effects of inhibition of basal nitric oxide synthesis on carotid-femoral pulse wave velocity and augmentation index in humans. Hypertension.

[31] Bellien J, Favre J, Iacob M, Gao J, Thuillez C, Richard V, Joannides R (2010). Arterial stiffness is regulated by nitric oxide and endothelium-derived hyperpolarizing factor during changes in blood flow in humans. Hypertension.

[32] Bergman RN, Ader M (2000). Free fatty acids and pathogenesis of type 2 diabetes mellitus. Trends Endocrinol Metab.

[33] Kabir M, Catalano KJ, Ananthnarayan S, Kim SP, Van Citters GW, Dea MK, Bergman RN (2005). Molecular evidence supporting the portal theory: a causative link between visceral adiposity and hepatic insulin resistance. Am J Physiol Endocrinol Metab.

[34] Zieman SJ, Melenovsky V, Kass DA (2005). Mechanisms, pathophysiology, and therapy of arterial stiffness. Arterioscler Thromb Vasc Biol.

[35] Arner P, Backdahl J, Hemmingsson P, Stenvinkel P, Eriksson-Hogling D, Naslund E, Thorell A, Andersson DP, Caidahl K, Ryden M (2015). Regional variations in the relationship between arterial stiffness and adipocyte volume or number in obese subjects. Int J Obes (Lond).

[36] Han SJ, Fujimoto WY, Kahn SE, Leonetti DL, Boyko EJ (2018). Change in visceral adiposity is an independent predictor of future arterial pulse pressure. J Hypertens.

