# Supplementary figures and images for: Visceral adiposity index is associated with arterial stiffness in hypertensive adults with normal-weight: the china H-type hypertension registry study
Source: Nutr Metab (Lond). 2021 Oct 9;18:90. doi: 10.1186/s12986-021-00617-5 (PMC8502385; doi:10.1186/s12986-021-00617-5)

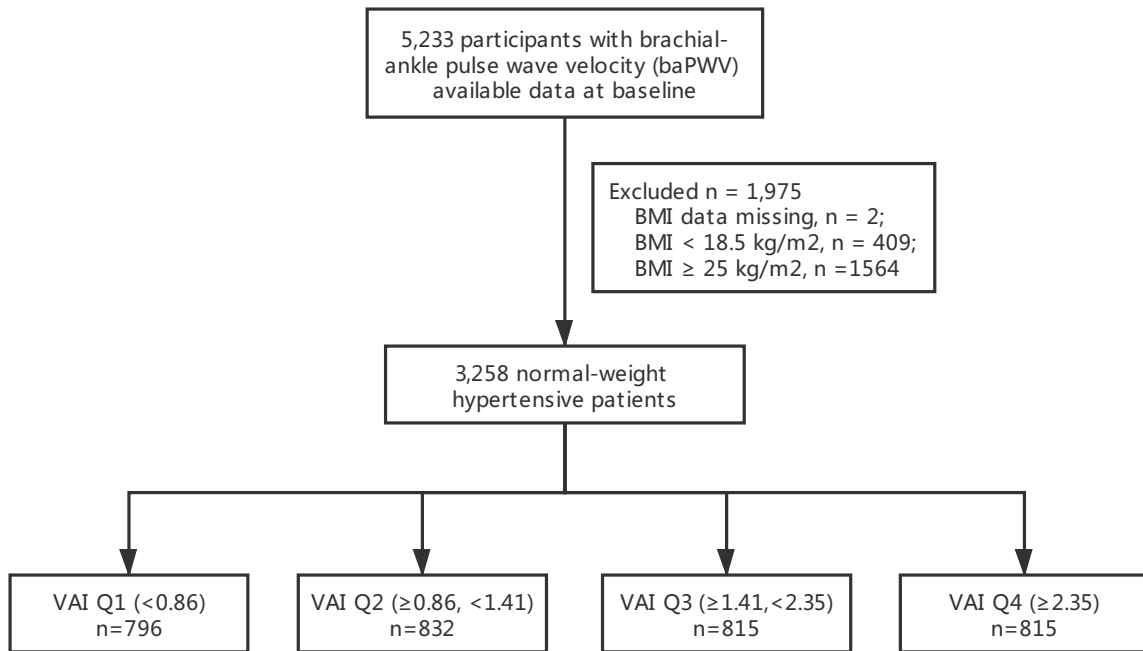

Supplement: Supplementary file 1 — Additional file 1. Figure S1. Flow chart of the study participants. [file 12986_2021_617_MOESM1_ESM.pdf]
